# High Resolution Niche Models of Malaria Vectors in Northern Tanzania: A New Capacity to Predict Malaria Risk?

**DOI:** 10.1371/journal.pone.0009396

**Published:** 2010-02-24

**Authors:** Manisha A. Kulkarni, Rachelle E. Desrochers, Jeremy T. Kerr

**Affiliations:** Canadian Facility for Ecoinformatics Research, Department of Biology, University of Ottawa, Ottawa, Ontario, Canada; INSERM U567, Institut Cochin, France

## Abstract

**Background:**

Malaria transmission rates in Africa can vary dramatically over the space of a few kilometres. This spatial heterogeneity reflects variation in vector mosquito habitat and presents an important obstacle to the efficient allocation of malaria control resources. Malaria control is further complicated by combinations of vector species that respond differently to control interventions. Recent modelling innovations make it possible to predict vector distributions and extrapolate malaria risk continentally, but these risk mapping efforts have not yet bridged the spatial gap to guide on-the-ground control efforts.

**Methodology/Principal Findings:**

We used Maximum Entropy with purpose-built, high resolution land cover data and other environmental factors to model the spatial distributions of the three dominant malaria vector species in a 94,000 km^2^ region of east Africa. Remotely sensed land cover was necessary in each vector's niche model. Seasonality of precipitation and maximum annual temperature also contributed to niche models for *Anopheles arabiensis* and *An. funestus* s.l. (AUC 0.989 and 0.991, respectively), but cold season precipitation and elevation were important for *An. gambiae* s.s. (AUC 0.997). Although these niche models appear highly accurate, the critical test is whether they improve predictions of malaria prevalence in human populations. Vector habitat within 1.5 km of community-based malaria prevalence measurements interacts with elevation to substantially improve predictions of *Plasmodium falciparum* prevalence in children. The inclusion of the mechanistic link between malaria prevalence and vector habitat greatly improves the precision and accuracy of prevalence predictions (r^2^ = 0.83 including vector habitat, or r^2^ = 0.50 without vector habitat). Predictions including vector habitat are unbiased (observations vs. model predictions of prevalence: slope = 1.02). Using this model, we generate a high resolution map of predicted malaria prevalence throughout the study region.

**Conclusions/Significance:**

The interaction between mosquito niche space and microclimate along elevational gradients indicates worrisome potential for climate and land use changes to exacerbate malaria resurgence in the east African highlands. Nevertheless, it is possible to direct interventions precisely to ameliorate potential impacts.

## Introduction

Malaria is the leading cause of death in African children, accounting for approximately 20% of all-cause mortality in children under the age of five [1]. Malaria transmission varies across Africa, a phenomenon that is well known but not easily predicted [2], [3], but can also vary dramatically between adjacent communities [4]. The heterogeneity of malaria transmission likely depends on environmental conditions that affect vector mosquito distributions, such as temperature, precipitation, humidity and land cover [5], which is further complicated by differences among the *Anopheles* vectors in their capacity to transmit the malaria parasite [6] and in vectors' responses to control strategies [7]. Ecological niche models use these environmental factors to predict the generalized distributions of malaria vectors across Africa [8], [9], [10], [11]. These models can be used to predict malaria risk continentally [12]. However, these models are too coarse to guide intervention efforts and their capacity to predict malaria prevalence remains uncertain.

Targeting limited intervention resources efficiently toward foci of malaria transmission would reduce malaria mortality and morbidity [4] but models lack the required precision to implement such strategies. Malaria transmission can vary considerably between households in the same community, depending on house construction or numbers of occupants, but environmental factors predict malaria risk at the community level [5]. Satellite remote sensing data have identified land covers [13], [14], [15] and topographical characteristics [16], [17] associated with vector breeding habitat and can be used to model malaria transmission [15], [16]. Accurate, high resolution ecological niche models for malaria vectors could bridge the spatial gap required to predict localized heterogeneity in malaria transmission and guide selection of locally appropriate vector control interventions.

The East African highlands are a frontier for potential resurgence of malaria due to climate change and malaria epidemics have become more common. The limits of malaria transmission in Africa are defined by the temperature-dependent development and survival of the vector and parasite [18], [19], which vary directly with altitude [20]. Warming climates could push malaria transmission to higher altitudes, resulting in more frequent epidemics among populations with little recent exposure to the disease [20], [21]. The link between altitude and malaria transmission is convincing [22], but differences in vector composition between humid coastal regions (where *An. gambiae* s.s. predominates) and arid interior regions (where *An. arabiensis* predominates) could also affect malaria transmission [8], [22]. The recent reemergence of *Plasmodium falciparum* epidemic malaria in the East African highlands is consistent with climate change [20], [23] but could also reflect other factors, such as antimalarial drug resistance [24], [25], land use changes [26], [27], or natural climate variability [28]. High resolution models predicting the distribution of malaria vectors and malaria transmission foci could help resolve the causes of malaria resurgence and serve as a basis for predicting where future malaria prevention and control efforts will be most needed.

Here, we develop high resolution measurements of environmental factors that previous research indicates should affect vector distributions in an area of northern Tanzania where recent land use and climate changes may be contributing to malaria resurgence. Our models have very high internal accuracies but, more importantly, they substantially improve predictions of malaria prevalence among children 2 to 9 years old, in terms of both accuracy (slope of observed vs. predicted prevalence does not differ from one) and precision (model r^2^ = 0.83). We demonstrate how validated models of malaria prevalence in East Africa, drawing on our high resolution vector niche models, can be used to map malaria risk with sufficient spatial detail (at 30×30 m resolution) to account for the extreme and localized heterogeneity in malaria transmission. These may provide a starting point for precise and potentially decisive interventions to reduce malaria risk in highly vulnerable populations.

## Methods

### Study Area

The study area ranged from areas west of Mount Kilimanjaro to the coastal plain of northern Tanzania, encompassing the Pare and Usambara Mountains. This area has a short rainy season in November and December and a long rainy season in March to May. Temperatures peak in January and are lowest in July. Malaria transmission is intense and perennial on the coastal plain [29] and moderate-to-low further inland [30], [31]. The three most common malaria vectors in Tanzania are *An. gambiae* s.s., *An arabiensis* and *An. funestus* s.l. [32]. *An. arabiensis* increases in the arid interior of Tanzania, while *An. gambiae* is concentrated in humid, coastal areas [33].

### Data

**Figure 1:**
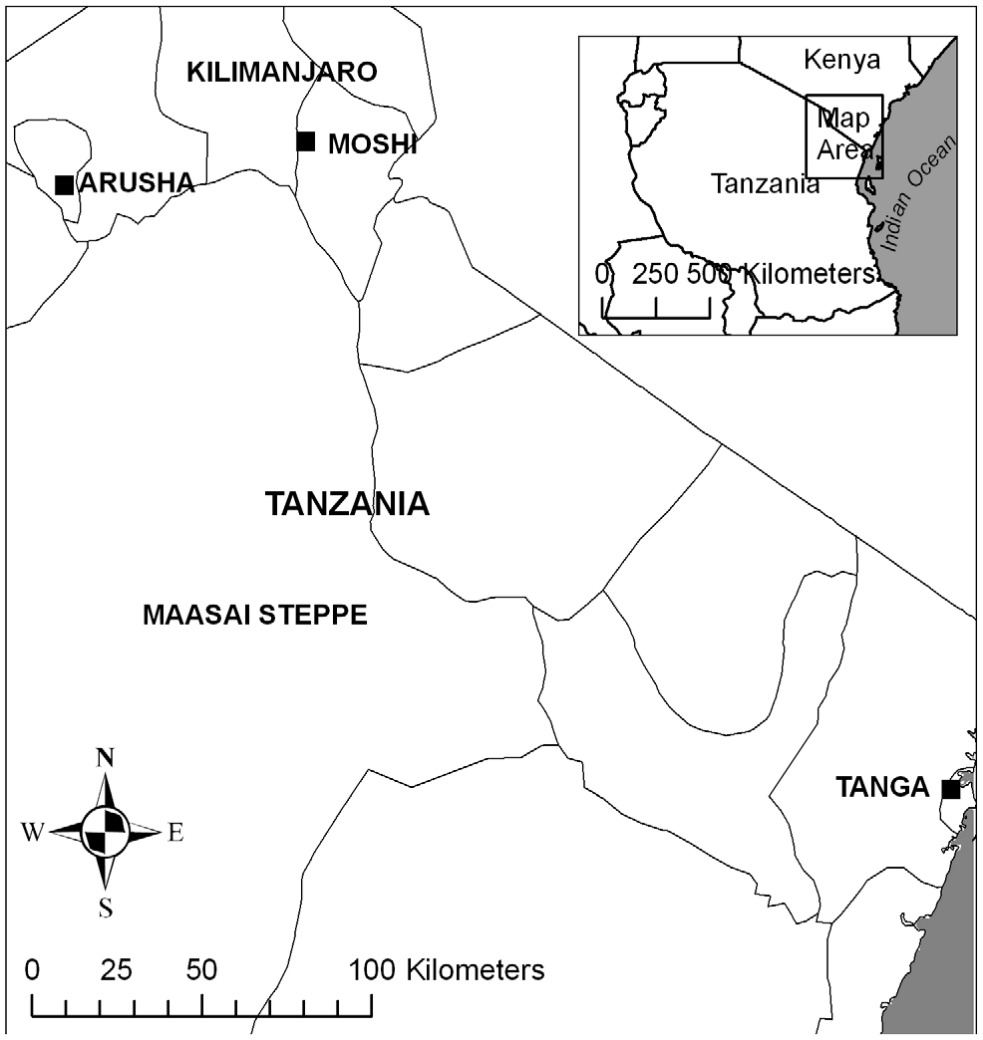
Map of study area in north eastern Tanzania.

Eight orthorectified Landsat 7 ETM+ scenes at 30-metre resolution, available for the years 2000 to 2002, were mosaicked to cover the area of northeastern Tanzania using Orthoengine in Geomatica v10.1 (PCI Geomatics, Richmond Hill, Canada). Nearly cloud-free scenes were available only during the dry season (January to early March), which served to reduce the potentially confounding influence of phenological differences and consequent classification errors along scene boundaries within the mosaic. Some haze, particularly along the eastern slopes of the Pare Mountains, was present, but was reduced by using the Haze Optimized Transform. The study area was clipped to 297 km ×317 km and covered large population gradients, from the densely population areas around Mount Kilimanjaro in the north-west and along the Indian Ocean coast in the south-east, and more sparsely populated areas in the arid interior (Figure 1).

Land cover was classified using Geomatica v10.1. Two image classification methods were used: (1) an unsupervised K-means classification with 50 statistically distinguishable clusters, corresponding to different vegetation types, and (2) a maximum-likelihood supervised classification with 8 pre-defined land cover classes (broadleafed evergreen forest, broadleafed deciduous woodland, rainfed crops, rice/irrigated crops, acacia scrubland, grassland, bare soil, water, and ice/snow) that were selected on the basis of likely biological significance for malaria vector habitat. For the supervised classification, 10–20 training polygons were created for each land cover type. This was facilitated through GPS ground-truthing across a ∼500 km east-west transect of the study area, including observation of land cover types at different elevations. The Africover database thematic classification for Tanzania [34] was cross-referenced to improve the accuracy of training site classification. To validate the classification scheme a set of 100 random sample points were compared between the known and classified land cover. Separability of land cover types was assessed by the Bhattacharyya distance, which measures the similarity of two discrete probability distributions. The classification output was assessed using the overall accuracy and the kappa statistic. Ascii grid layers for unsupervised and supervised land cover classifications were exported to ArcGIS v9.2 (ESRI, Redlands, CA) for further manipulation.

Nineteen environmental layers were obtained from the WorldClim database, including 11 temperature and 8 precipitation indices that express spatial variation in annual means, seasonality and extreme climatic factors [35]. Each layer was available at a resolution of 30 arc-seconds (∼1 km) and was resampled to 1 arc-second resolution. Resampled environmental layers were clipped to match dimensions of the landcover classification grids then transformed to ascii grids. Elevation data from the Shuttle Radar Topography Mission (SRTM) at a resolution of 3 arc-seconds (90 metres), and human population density for the year 2006, from the Oak Ridges National Laboratory Landscan database (http://www.ornl.gov/landscan), available at 30 arc-second resolution (∼1 km), were similarly processed.

Occurrence records for *An. arabiensis*, *An. gambiae* s.s. and *An. funestus* s.l. were obtained from field collections conducted between 2001–2004 [36] and supplemented by a review of published literature sources and unpublished data sources provided by local experts. Inclusion criteria for model development were: collection of adult specimens in 2000 or later, morphological identification of *An. funestus* s.l., identification of *An. gambiae* s.l. to sibling species level by polymerase chain reaction or cytogenetics, and village-level georeferencing. Sites where both *An. gambiae* s.s. and *An. arabiensis* were reported were allocated to the predominant sibling species if it represented >80% of vectors collected, otherwise the site was included for both species. Eighteen records met the inclusion criteria for *An. arabiensis* while only 7 and 10 records were retained for *An. gambiae* s.s. and *An. funestus* s.l., respectively. To overcome the scarcity of geographically distinct records during the desired collection period, the dataset was supplemented with records from earlier studies by Mnzava et al. (1995) [37] and Mnzava and Kilama (1986) [33]; village coordinates for these records were obtained from the Global Biodiversity Information Facility database (http://www.gbif.org). In total 29 geographically distinct records were used for model development, providing occurrence data for *An. arabiensis* (n = 20), *An. gambiae* s.s. (n = 10) and *An. funestus* s.l. (n = 10) (Figure 1).

Malariometric data was obtained from two cross-sectional surveys conducted in 2001 by Drakeley et al. (2005) in 24 villages in the Kilimanjaro and Tanga regions [22]. Study sites were selected by the authors to cover large environmental and altitudinal gradients while minimizing socioeconomic differences. The prevalence of alleles associated with resistance to sulfadoxine-pyrimethamine, the first line treatment in 2001, was found to be similar across the study area in northern Tanzania [38], [39]. In the present study, the mean prevalence of *Plasmodium falciparum* from the short and long rainy season surveys was used as an indicator of malaria transmission to reduce seasonal variability. Analysis was restricted to malaria prevalence in children aged 2–9 years to control for the confounding effect of infant immunity on parasitemia [40] and the potential effect of increased population movement and/or acquired immunity in older age groups.

Shapefiles of administrative boundaries such as water bodies and rivers were used only for mapping purposes and were obtained from the FAO GeoNetwork database.

### Niche Models

Maximum entropy (Maxent) software [41] was used to predict the distribution of each species over the chosen geographical region. Maxent uses presence-only occurrence data in conjunction with environmental data to predict areas that target species may occupy. Maxent models are sometimes more conservative than alternative machine-learning techniques for predicting species potential distributions [12]. However, comparative studies have demonstrated that Maxent is among the most reliable species distribution modeling techniques and retains its effectiveness well even when species have been observed in only a small number of localities [42], [43].

To limit the influence of individual occurrence records on the overall model and to provide a means for assessing model accuracy, data were randomly partitioned for model evaluation, with 75% of the records used as training data to construct the models and the remaining 25% set aside for testing. The accuracy of each model was determined by performing both a threshold-dependent binomial test of omission and a threshold-independent receiver operating characteristic analysis [41]. For the binomial test of omission a threshold of 0.1 was selected from the output generated by Maxent; a p-value <0.05 was used to indicate whether the niche model outperformed a random model [41]. For the threshold-independent receiver operating characteristic analysis, which produces a curve of sensitivity vs. 1-specificity, only models with an area under the curve (AUC) greater than 0.90 were retained. The AUC can be used as an overall estimate of the model's discriminating ability, usually expressed as accuracy. AUC values range between 0 and 1, with maximum accuracy achieved with values of 1, accuracy no better than random with values of 0.5 and values <0.5 indicating performance worse than random. Similar protocols for model retention have been used previously [12]. A jackknife procedure was used to determine the contribution of each variable to the model. Maxent calculates the AUC of the model using each parameter individually, and again after omitting each of the parameters one at a time [41]. Those parameters for which the difference between these values is highest can be interpreted as possessing the most information not present in the other variables. Based on this analysis, the contribution of parameters to the model is determined as a percentage.

A two-step selection procedure was used to develop niche models for individual vector species. Separate covariance matrices were first generated for the 11 temperature and 8 precipitation bioclimatic variables; variables were selected that minimized covariance and represented biologically important criteria for vector species. Two models were then constructed for each species, using either the supervised or the unsupervised land cover classification as a categorical variable in addition to the relevant bioclimatic variables, elevation and human population density as continuous variables. Accuracy assessments were applied to evaluate the goodness-of-fit of each model. Ten model replicates were run for each species, each with a random partitioning of training and test data, and the raster maps of probability of suitability output by Maxent were averaged to determine the probability of suitability for each grid cell.

The averaged probability of suitability map for each species was converted into a binary map of predicted suitable and non-suitable areas. A decision threshold was defined for each model, above which the habitat suitability for the species was considered to be high and below which the habitat suitability was considered to be low. For each species, the average predicted suitability of training records that were used to construct the model was applied as the threshold value. This is considered an effective method to maximize both sensitivity and specificity, i.e. minimize false negative and false positive rates [44]. A cumulative (all species) grid of predicted vector habitat was created from the binary species grids. To this end, cell values were set to one where the value of the summed individual species grids was greater than or equal to one to reflect areas of suitable habitat for any vector species, while cells with zero value reflected areas where the habitat was unsuitable for any vector species.

### Statistical Analysis

The strong association between proximity to breeding sites and adult vector abundance has been demonstrated in different environments across Africa [15], [45], [46]. Bogh et al. (2007) found that the distance-weighted area of malaria vector breeding habitat near villages in The Gambia was strongly associated with vector density and was a reliable predictor of malaria transmission. Therefore, to relate village-level malaria prevalence to predicted vector species' habitat, a buffer of 0.012 decimal degrees (∼1.5 km) was created around each village, reflecting the potential dispersal range of Afrotropical *Anopheles* mosquitoes [47]. The mean value of cells within the buffer zone was sampled from the binary habitat grids to obtain the proportion of area suitable for vector species (individual and cumulative) within a radius of 1.5 km of each village.

Ordinary least squares (OLS) regression was used to test for the effect of altitude and cumulative vector species habitat on *P. falciparum* prevalence in children using proc GLM in SAS v9.1 (SAS Institute, Cary, NC). Arcsine square-root transformations were applied to proportional data. The model residuals were tested for spatial autocorrelation and the associated Moran's I value calculated using SAM v3.0 [48]. Conditional autoregressive (CAR) models were constructed to adjust for residual spatial autocorrelation and coefficients compared with those from the OLS regression model, where necessary. Model goodness-of-fit was assessed by linear regression of observed versus predicted malaria prevalence.

Regional differences in village-level vector species' habitat were compared using a non-parametric two-way ANOVA with interaction term. A Kruskal-Wallis rank sum test was used to test for significance.

## Results

### Land Cover Classification

The K-means unsupervised land cover classification generated 50 statistically distinct clusters; the resulting land cover map was applied in Maxent models in comparison with the supervised land cover classification. The overall accuracy of the maximum likelihood supervised land cover classification was 95%, with a kappa coefficient of 0.98. The separability of pairs of land cover types was measured by the Bhattacharyya distance. The minimum separability was found between broadleafed evergreen forest and broadleafed deciduous forest land cover types. This is likely of minor importance for the prediction of vector habitat because both land cover types have low biological suitability for the malaria vector species of interest. However, below average separability was also measured between rice/irrigated agriculture and broadleafed deciduous forest, and between acacia scrubland and rainfed crops, which could affect the prediction of vector habitat due to the importance of these land cover types for vector species in Tanzania.

### Niche Models

Unsupervised land cover classification outperformed the supervised classification of land cover types in the niche models for all species, as indicated by the AUC and percentage contribution of individual variables (Table 1). Niche models therefore retained the unsupervised land cover classification to generate binary habitat suitability maps. Precipitation seasonality (measured as the coefficient of variation of monthly precipitation amount), maximum temperature of the warmest month and land cover contributed the most to the niche models for *An. arabiensis* and *An. funestus* (Table 2). The maximum training gain for both species from jackknife analysis was associated with precipitation seasonality, indicating that this variable contained the most information not present in other variables. In contrast, precipitation of the coldest quarter (June to August, also the driest quarter), land cover and altitude contributed the most to the niche model for *An. gambiae* s.s. and the maximum training gain for this species was associated with precipitation of the coldest quarter.

**Table 1:** Comparison of malaria vector species' niche models using unsupervised vs. supervised land cover classifications.

	Land cover classification	
Species	Unsupervised	Supervised
*An. arabiensis*		
Model AUC	0.989	0.979
% contribution to model	14.9	2.8
*An. funestus*		
Model AUC	0.991	0.958
% contribution to model	25.7	14.3
*An. gambiae*		
Model AUC	0.997	0.989
% contribution to model	15.0	3.0

**Table 2:** Contribution of environmental variables to malaria vector species' niche models.

Species	Variable	% Contribution to Model	AUC without variable	AUC with only variable	Model AUC
*Anopheles arabiensis*	precipitation seasonality*	48.5	0.983	0.866	0.989
	maximum temperature of the warmest month	16.6	0.990	0.766	
	land cover (unsupervised)+	14.9	0.979	0.889	
	human population density	9.2	0.985	0.776	
	precipitation of the warmest month	4.1	0.990	0.763	
	temperature annual range	3.9	0.989	0.736	
	temperature seasonality	1.9	0.989	0.764	
	precipitation of the coldest month	0.8	0.989	0.531	
	altitude	0.1	0.990	0.570	
*Anopheles funestus*	maximum temperature of the warmest month*	37.2	0.991	0.826	0.991
	precipitation seasonality	35.7	0.963	0.833	
	land cover (unsupervised)+	25.7	0.940	0.951	
	temperature seasonality	0.9	0.987	0.598	
	human population density	0.5	0.988	0.731	
	Altitude	0.0	0.987	0.595	
*Anopheles gambiae*	precipitation of the coldest quarter* +	64.7	0.997	0.974	0.997
	land cover (unsupervised)	15.0	0.990	0.949	
	Altitude	8.5	0.997	0.951	
	minimum temperature of the coldest month	5.3	0.997	0.923	
	annual precipitation	2.1	0.997	0.959	
	mean temperature of the warmest quarter	1.6	0.997	0.944	
	maximum temperature of the warmest month	1.0	0.997	0.910	
	precipitation of the driest quarter	1.0	0.997	0.967	
	precipitation of the warmest quarter	0.3	0.997	0.640	
	human population density	0.4	0.997	0.864	

*largest % contribution to model.

+ largest AUC on its own.

**Figure 2:**
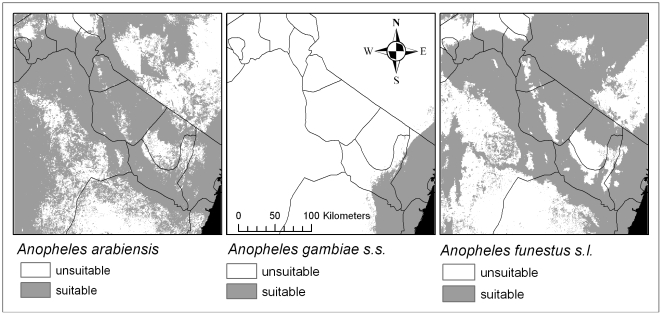
Malaria vector niche models. Models show the area of predicted suitable habitat (shaded area) at a resolution of 30×30 metres across north eastern Tanzania for the dominant malaria vector species, *An. arabiensis*, *An. gambiae* s.s. and *An. funestus* s.l.

Modelled habitat suitability differed substantially between the three vector species (Figure 2). Models predicted *An. arabiensis* habitat to be most widely distributed, including in highland areas to an altitude of 2300 metres. This species' habitat is predominantly in lowland areas associated with river valleys and large-scale irrigated rice production. In contrast, *An. gambiae* s.s. habitat is concentrated in coastal plains, but our models suggest that this species may extend its range up to 1200 metres in the Eastern Usambara mountains. *An. funestus* s.l. habitat is more restricted than *An. arabiensis* and is not predicted to occur in areas higher than 1900 metres, although the two species are predicted to be sympatric over much of the study area.

The mean proportion of suitable habitat surrounding villages differed for vector species (P<0.0001). For villages in the Kilimanjaro region, the mean proportion of suitable habitat within a radius of 1.5 km of the village was 52% (95%CI 0.28–0.76) for *An. arabiensis*, 38% (95%CI 0.08–0.68) for *An. funestus* s.l. and 0% for *An. gambiae* s.s. For villages in the Tanga region, the mean proportion of suitable habitat was 39% (95%CI 0.21–0.58) for *An. arabiensis*, 25% (95%CI 0.0–0.51) for *An. funestus* s.l. and 18% (95%CI 0.0–0.40) for *An. gambiae* s.s. Differences in the proportion of suitable vector habitat around villages in the two regions were not statistically significant (P = 0.730), although biologically significant patterns were observed with the highest concentration of An. gambiae s.s. habitat in the Tanga region.

### Statistical Analysis

**Figure 3:**
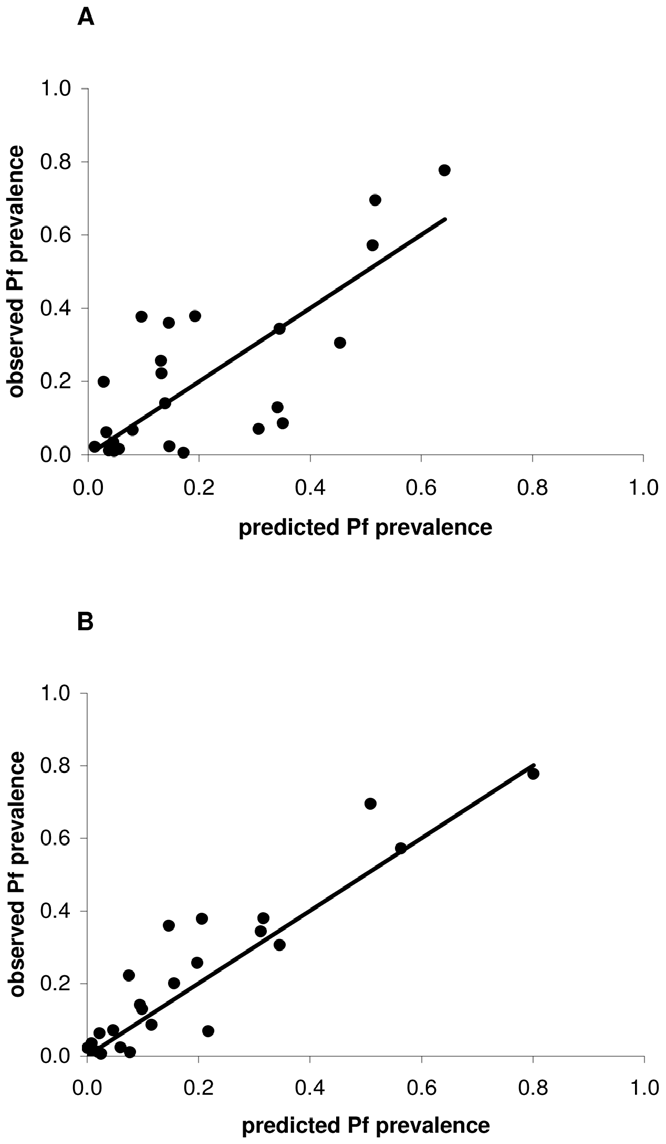
Regression model goodness-of-fit. Area of predicted malaria vector habitat improves the goodness-of-fit of models of malaria prevalence, assessed by regression of observed versus predicted malaria prevalence in children 2 to 9 years old. Assessments were performed for (A) an ordinary least squares regression model of *P. falciparum* prevalence as a function of altitude, and (B) a conditional autoregressive model of *P. falciparum* prevalence as a function of altitude and habitat. Data points represent 24 villages in north eastern Tanzania. The 1:1 line is shown for reference.

Altitude relates strongly to *P. falciparum* prevalence, but model residuals showed significant spatial autocorrelation at distances less than 50 km (Moran's I = 0.366, P = 0.006). A conditional autoregressive model was constructed to minimize potential influences of spatial autocorrelation on probability tests and model parameters (r^2^ = 0.50, P<0.001). The relationship between mean parasite prevalence and altitude was described by the equation:
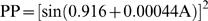
where PP is the prevalence of *P. falciparum* in children 2 to 9 years old and A is the altitude in metres from SRTM data. The regression of predicted malaria prevalence versus observations, while reasonably accurate (r^2^ = 0.56), demonstrated a non-linear trend that could bias predictions of malaria prevalence (Figure 3).

Multiple linear regression revealed a significant interaction between altitude and habitat on *P. falciparum* prevalence in children that substantially improved model accuracy (model r^2^ = 0.828, P<0.001), as well as significant main altitude and non-significant main habitat effects. Area of vector habitat around villages affects malaria prevalence differently with altitude. 3D plots of the altitude-habitat interaction effect on parasite prevalence reveal that habitat is least important at the highest and lowest elevations and of greatest importance at mid-altitudes (i.e. the slope of the interaction term is steepest there). The model residuals were not significantly autocorrelated at any distance class (Moran's I = 0.217, P>0.05) and coefficients were nearly identical to those derived from a CAR model, consistent with the absence of residual spatial autocorrelation. Addition of area of predicted vector habitat to the model thus accounted for 65.6% of the previously unexplained variance in prevalence of *P. falciparum* in young children. The linear relationship between predicted malaria prevalence and prevalence observations indicates unbiased model prediction with improved precision (r^2^ = 0.86; slope = 1.021, 95% CI 0.987–1.056; intercept = 0.026, 95% CI 0.017–0.036) (Figure 3).

**Figure 4:**
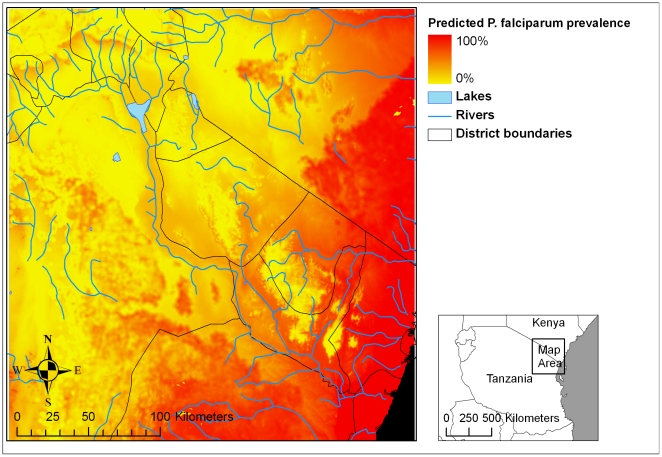
Continuous map of predicted malaria prevalence. Predicted *P. falciparum* prevalence in children 2 to 9 years old as a function of altitude and vector habitat availability within 1.5 km of grid cells (predicted from niche models) is shown at 30×30 metre resolution on a continuous scale.

**Figure 5:**
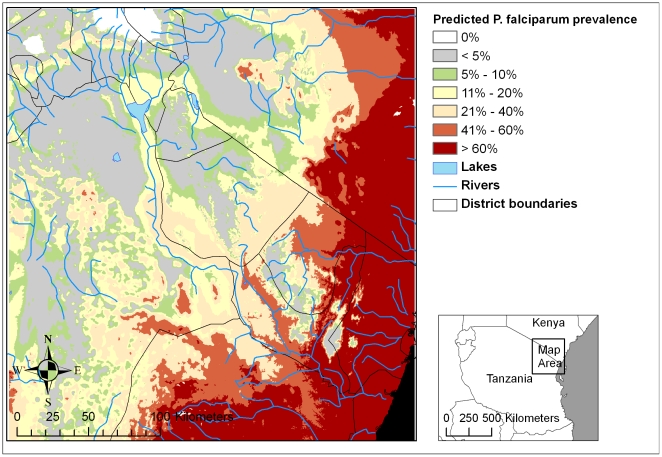
Categorical map of predicted malaria prevalence. Predicted *P. falciparum* prevalence in children 2 to 9 years old as a function of altitude and vector habitat availability within 1.5 km of grid cells (predicted from niche models) is shown on a categorical scale.

The relationship between mean parasite prevalence, altitude and area of predicted vector habitat around villages was described by the equation:
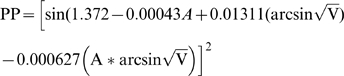
where A is the altitude in metres from SRTM data and V is the proportion of area suitable for malaria vector habitat within a radius of 1.5 km of each 30 m grid cell, predicted from the cumulative vector species niche model. This equation was used to generate a map of predicted malaria risk throughout the study region at a resolution of 30×30 m (Figure 4). Parasite prevalence was considered negligible in cells that had no suitable vector habitat in the surrounding 1.5 km radius. Categories were generated to reflect meaningful levels of malaria endemicity (Figure 5).

## Discussion

Satellite-derived data is being increasingly employed to generate maps and predictive models of malaria risk, usually at a global or continental scale [3], [8], [10], [12]. While these maps provide useful indications of the distribution and dynamics of malaria vectors and/or malaria transmission across the African continent, and may be used to project large-scale changes in malaria distribution under different climate change scenarios [10], [49], they are of limited operational use for targeting malaria control interventions [4]. A recently published contemporary global map of malaria endemicity is expected to facilitate regional planning of malaria control in endemic countries [3], but it lacks a mechanistic link to vector mosquito habitat that could improve the accuracy and spatial resolution of predictions, particularly in areas with sparse malaria prevalence data.

Although ecological niche models constructed using Maximum Entropy are considered relatively reliable, few studies are able to use independent data to test whether those models provide effective biological insight [50]. Here, we demonstrate that not only are niche models for competent vectors for malaria highly accurate based on internal resampling (AUC>0.9), but those niche models independently improve the best previous prediction for malaria prevalence among children 2 to 9 years old by 65.6%. The inclusion of spatially detailed habitat characteristics, as assessed by the detailed multispectral assessment possible using Landsat Thematic Mapper data, in addition to coarser resolution climatic data, provides tactically useful guidance around the distribution of malaria vector species. Areas with higher seasonality of precipitation were predicted to be most suitable for *An. arabiensis*, while areas that experienced greater precipitation in the cold, dry season favoured *An. gambiae* s.s. This is consistent with the adaptation of the former sibling species to withstand seasonal aridity, while the more competent vector, *An. gambiae* s.s., is best adapted to areas that experience year-round humidity. Suitability for *An. arabiensis* was further seen to peak in areas with moderate human population densities while low suitability was observed in areas with very sparse or very dense populations, suggesting a reduction of suitable habitat in heavily populated, urban areas. While urbanization was not explicitly addressed in our analysis, this suggests the importance of including human population density in vector species distribution models. An important implication of our ecological niche models is that the predicted distribution of malaria vector species in northern Tanzania can inform the selection of locally appropriate vector control interventions. Our models suggest that *An. arabiensis* is most widely distributed in the northern Tanzanian interior. This implies reduced efficacy of indoor residual spraying (IRS) as the preferred strategy for mitigation of malaria epidemics in highland areas [51], because this exophilic vector is less susceptible to indoor applications of residual insecticides in contrast to *An. gambiae* s.s. and *An. funestus* s.l., which remain indoors after feeding. History has shown that the success of IRS programs may be jeopardized in areas with vector species combinations [52].

This study develops land cover data intended to reveal ecological differences important for predicting malaria vector species distributions by means of an unsupervised classification of high resolution Landsat 7 ETM+ data. The resulting land cover led to large improvements in malaria prevalence prediction by contributing to prediction of vector habitat distribution, probably by successfully detecting potential vector breeding habitat. Previous land cover data for the region were highly aggregated and do not improve predictions of malaria prevalence. The diminished thematic detail of generalized land cover data at coarser resolutions, such as well-known global and continental products from the Advanced Very High Resolution Radiometer (AVHRR) or Moderate Resolution Imaging Spectroradiometer (MODIS) data [53], are also less likely to provide the necessary biological insights to distinguish malaria vector habitat. The use of land cover data that were not developed to assess habitat suitability for particular species may explain why land cover is often found to be an ineffective addition to niche models [12]. We show that detailed assessment of vector habitat, measured here as the area of vector species' habitat within a radius of 1.5 km of villages, improves models of malaria prevalence substantially relative to previous work demonstrating the primary importance of altitude. The spatial detail present in such models, as well as their demonstrated effectiveness in predicting disease prevalence, permits high resolution mapping of malaria risk in an epidemic-prone region in Africa which may improve the effectiveness of malaria control strategies. These mapping efforts may further provide a baseline to evaluate the impact of malaria interventions.

Our analysis suggests that vector habitat exerts an important influence on malaria prevalence at mid-altitudes, but that its influence at altitudinal extremes diminishes. At very low altitudes this could reflect the saturation of infection with increasing transmission, as measured by the entomological inoculation rate (EIR) [54]. Host immunity, antimalarial drug use, or coverage with insecticide-treated nets could also affect malaria prevalence at low altitudes, diminishing the importance of vector habitat as a predictor. Our model may provide a means to assess the relative importance of these factors to site-specific malaria prevalence, recognizing the primary importance of environmental factors. At very high altitudes, temperature-dependent effects on parasite survival and development may limit malaria transmission despite the presence of suitable vector habitat. This phenomenon, referred to as ‘anophelism without malaria’, while common in Europe and North America [55], has not previously been described from the East African highlands. Rather, despite the presence of suitable niche characteristics, low vector densities likely limit malaria transmission in the East African highlands. Epidemics follow weather that favours formation of productive larval habitats and that accelerates larval development, namely increased temperatures and rainfall [56], [57]. Increases in transmission may be highly localized, and influenced to a large extent by topography, as observed by Lindsay et al. [58] following an El Nino event. Our modelling approach may identify areas at increased risk of epidemics if seasonal weather fluctuations and/or broader warming trends create favourable conditions for parasite transmission.

Despite the significant contribution of predicted vector species habitat to models of malaria transmission, this study has several assumptions. First, we were unable to test the predictive ability of our malaria prevalence model using independent data. Insufficient community-based prevalence data, collected using similar controlled methodology, were available within the study area to provide the basis for independent testing. This highlights the need for systematic collection of community-based measures of malaria prevalence in Tanzania and more broadly across the African continent. Second, we assume that adult occurrence records represent areas of suitable vector habitat. Given that breeding sites are often located within several hundred metres of sites with abundant adult vectors [15], [45], [46], and that land cover clusters tend to extend over multiple grid cells, this is unlikely to affect the accuracy of our niche models at 30 metre resolution. In addition, while the entomological dataset represents the most comprehensive and contemporary set of vector species occurrence records available for northern Tanzania, the data span a period of almost 10 years. Geographic instability in vector occurrences over this period could affect the accuracy of the resulting models. Fortunately, this effect would limited by the data partitioning and use of test replicates in the construction of niche models. Finally, the correlation between satellite data and vector abundance has been well described for other insect vectors such as tsetse flies, which transmit African trypanosomiasis [59], [60] and have relatively stable population dynamics. The relationship between malaria vector habitat and vector abundance, however, is more complex and temporally variable because of seasonality of temperature and rainfall [46], [57]. Lack of predicted habitat suitability for *An. gambiae* s.s. in the Southern Pare Mountains, where low proportions (5–10%) of this sibling species have been described relative to *An. arabiensis* [33], [61], suggest our niche model for *An. gambiae* s.s. may be conservative in areas of limited or seasonal species occurrence. Our simplified use of a cumulative index of vector species habitat is expected to eliminate inaccuracies in the spatial prediction of malaria prevalence resulting from seasonal variation in vector species composition. Analysis with multi-temporal datasets could reveal seasonal contributions of individual vector species to transmission and further allow for weighting to reflect inherent differences in vectorial capacity. This would be of interest if the altitude-independent differences in malaria transmission observed by Drakeley et al. (2005) in part relate to a seasonal succession of different vector species, which serves to extend the transmission season in areas with sympatric vectors, such as the coastal zone in Tanzania. Ecological niche modelling of seasonal vector population dynamics is an important area of future research.

By combining ecological niche models with purpose-built, high resolution satellite remote sensing data, models predicting malaria prevalence in children improve dramatically. This improvement takes two forms: statistical precision increases by 65.6% and prediction bias disappears (observed versus predicted prevalence has a slope of 1 and an intercept of 0). It is clear that ecological niche models can provide strong benefits for malaria risk mapping when exhaustive field characterization of vector breeding sites is impractical, particularly when detailed remote sensing data are included. Maps such as these can inform the selection of locally appropriate control strategies based on vector species assemblages and identify potential foci of transmission to better target scarce malaria control resources. With the availability of high resolution multi-temporal land cover data, our model could be applied to detect seasonal range expansions/contractions for individual vector species; this is currently not feasible using Landsat TM data, for which few cloud-free scenes are available. Importantly, recent malaria vector species niche models for the African continent suggest the expansion of vector habitat in Eastern and Southern Africa may result from climate change [49]. Projection of high resolution niche models using general circulation model (GCM) scenarios could contribute to the understanding of potential future effects of climate change on local malaria vector distributions in the East African highlands.
